# Single-cell transcriptome analysis reveals liver injury induced by glyphosate in mice

**DOI:** 10.1186/s11658-023-00426-z

**Published:** 2023-02-04

**Authors:** Jiangpeng Wu, Xiuping Sun, Chunyi Wu, Xiaoping Hong, Lulin Xie, Zixu Shi, Liang Zhao, Qingfeng Du, Wei Xiao, Jichao Sun, Jigang Wang

**Affiliations:** 1 Department of Nephrology, Shenzhen Key Laboratory of Kidney Diseases, and Shenzhen Clinical Research Centre for Geriatrics, Shenzhen People’s Hospital (The Second Clinical Medical College, Jinan University, The First Affiliated Hospital, Southern University of Science and Technology), Shenzhen, 518020 China; 2 Department of Rheumatology and Immunology, Shenzhen People’s Hospital (The Second Clinical Medical College, Jinan University, The First Affiliated Hospital, Southern University of Science and Technology), Shenzhen, 518020 China; 3 Key Laboratory of Glucolipid Metabolic Disorder, Ministry of Education, Guangdong Pharmaceutical University, Guangzhou, 510006 China; 4 School of Traditional Chinese Medicine, Southern Medical University, Guangzhou, 510515 China; 5 Dongguan Maternal and Child Health Care Hospital, Postdoctoral Innovation Practice Base of Southern Medical University, Dongguan, 523125 Guangdong China; 6 Department of Pathology, Shunde Hospital, Southern Medical University (The First People’s Hospital of Shunde), Foshan, 528300 China; 7 Department of Pathology and Guangdong Province Key Laboratory of Molecular Tumor Pathology, School of Basic Medical Sciences, Southern Medical University, Guangzhou, 510515 China; 8 Artemisinin Research Center, and Institute of Chinese Materia Medica, China Academy of Chinese Medical Sciences, Beijing, 100700 China

**Keywords:** Glyphosate, Hepatotoxicity, scRNA-seq

## Abstract

**Background:**

Glyphosate (GLY), as the active ingredient of the most widely used herbicide worldwide, is commonly detected in the environment and living organisms, including humans. Its toxicity and carcinogenicity in mammals remain controversial. Several studies have demonstrated the hepatotoxicity of GLY; however, the underlying cellular and molecular mechanisms are still largely unknown.

**Methods:**

Using single-cell RNA sequencing (scRNA-seq), immunofluorescent staining, and in vivo animal studies, we analyzed the liver tissues from untreated and GLY-treated mice.

**Results:**

We generated the first scRNA-seq atlas of GLY-exposed mouse liver. GLY induced varied cell composition, shared or cell-type-specific transcriptional alterations, and dysregulated cell–cell communication and thus exerted hepatotoxicity effects. The oxidative stress and inflammatory response were commonly upregulated in several cell types. We also observed activation and upregulated phagocytosis in macrophages, as well as proliferation and extracellular matrix overproduction in hepatic stellate cells.

**Conclusions:**

Our study provides a comprehensive single-cell transcriptional picture of the toxic effect of GLY in the liver, which offers novel insights into the molecular mechanisms of the GLY-associated hepatotoxicity.

**Supplementary Information:**

The online version contains supplementary material available at 10.1186/s11658-023-00426-z.

## Background

Glyphosate (GLY) is an active ingredient of the world’s most widely used herbicide, which has broad-spectrum activity for weed control in agriculture [1, 2]. Its usage has dramatically increased since the introduction of genetically modified GLY-resistant crops [2, 3]. Due to its wide usage, GLY is commonly detected in plants, soils, water, air, animals, and humans [4–8].

By inhibiting the 5-enolpyruvylshikimate-3-phosphate synthase enzyme, GLY blocks the synthesis of aromatic amino acids via the shikimate pathway, which exists in plants, bacteria, fungi, and protozoa but not in mammals [9, 10]. Hence, GLY was considered relatively safe for human beings by several reports and regulatory authorities such as the European Food Safety Authority [11, 12]. However, this opinion has been challenged because accumulated studies have disclosed the potential health risks of GLY. GLY could cause damages in liver and kidney even at concentrations below the acceptable daily intake as defined by German Federal Institute for Risk Assessment [13, 14]. The doses of GLY exposure are significantly correlated with the stages of liver fibrosis in patients [15]. Upon GLY exposure, rat and mouse livers display abnormal liver function indicated by liver panel test, and dysregulated lipid metabolism by chemical biology study [16–18]. As for in vitro studies, GLY-based herbicides resulted in DNA damage and endocrine disruption in human cell lines—HepG2 and MDA-MB453-kb2—under sub-agriculture doses [19]. GLY induced proliferation of T47D cells, a human hormone-dependent breast cancer cell line [20]. Besides, the adjuvants used to enhance water solubility and plant absorption of GLY are often detected in the environment and induce cell toxicity [21–23]. It was not quite clear how GLY degrades in mammals, but its metabolites such as aminomethylphosphonic acid (AMPA) were detected after GLY exposure in animals [24–26] and humans [27]. It has been reported that AMPA, the primary metabolite of glyphosate, could also introduce toxicity [28–31]. These concerns led to the reclassification of GLY as a probable carcinogen (Group 2A) by the International Agency Research on Cancer [32]. Moreover, approximately 20 countries, including France, Belgium, Italy, and Thailand, have either restricted or banned the usage of GLY [33].

As the most important detoxification organ, the liver plays a pivotal role upon toxic substance exposure. Though studies have revealed the potential hepatotoxicity of GLY, how GLY influences specific cell types in liver remains unclear. The advent of single-cell RNA sequencing (scRNA-seq) technology made it possible to profile the gene expression at single-cell level in complex tissues. Here, we performed scRNA-seq of the liver tissues from mice exposed to GLY. GLY induced changes in cell composition, transcription, and intercellular communication, and exerted hepatotoxicity effects by affecting specific cell (sub)types and corresponding genes. Specifically, GLY induced dysfunctions in hepatocytes by abnormal lipid metabolism, inflammatory response, and DNA damage, and in Kupffer cells (KCs) through enhanced reactive oxygen species (ROS) production, oxidative stress response, and phagocytosis. GLY exposure also induced activation of proliferation and extracellular matrix production in hepatic stellate cells (HSCs). Moreover, liver capsular macrophages (LCMs) were activated after GLY exposure as reflected by the significantly increased cell proportion and upregulation of phagocytosis, endocytosis, and inflammatory response. Collectively, our findings provide a comprehensive single-cell transcriptional picture of the toxic effect of GLY in the liver, which offers novel insights into the molecular mechanisms of the GLY-associated hepatotoxicity.

## Methods

### Chemicals

GLY (chemical purity 99.5%; CAS: 1071-83-6) was purchased from Shanghai Aladdin Biochemical Technology Co., Ltd. (Shanghai, China, P109919-250 mg).

### Animals and experiment design

Seven-week-old male C57BL/6J mice, weighing around 20 g, were purchased from GemPharmatech (Guangdong, China) and housed in plastic cages with a layer of sawdust to adapt to the environment for 7 days. Throughout the experimental period, the mice were maintained in colonies at 22 ± 2 °C, on a 12 h light/dark cycle, with controlled humidity (50 ± 5%) and free access to standard laboratory mouse diet and water. The cages were periodically rotated to reduce effects caused by cage position. The mice were randomly allocated to two groups, each with three mice, followed by intraperitoneal injection with PBS (vehicle control) or 200 mg/kg GLY (10 mL/g mouse weight) once per day for 7 days, respectively. The dosage was selected on the basis of a previous toxicity study [18]. The mice were sacrificed by cervical dislocation and perfused with PBS for tissue collection.

### Histological examination and statistical analysis

A portion of the liver was fixed by 4% paraformaldehyde for 1 day at 4 °C and then embedded in paraffin. After dissection into slices, hematoxylin and eosin (H&E) staining was conducted and slices were observed using a microscope. The statistical analysis of the degree of necrosis was conducted on the basis of the H&E staining images. Numerical assessment numbers 0, 1, 2, 3, and 4 represent none, single-cell necrosis, no more than 30%, no more than 60%, and more than 60%, respectively.

### scRNA-seq data analysis

Raw single-cell sequencing data were processed by the Cell Ranger pipeline (version 6.0.1) with recommended parameters and aligned to the mm10 mouse transcriptome for quantification of transcripts in each cell. The pipeline analysis generated a gene expression matrix for each sample, which contained barcoded cells and gene expression counts. The gene-barcode matrix was loaded into the Seurat package (version 4.0.3) for further downstream analyses using R toolkit. The possible low-quality or dual-nucleated cells were filtered and excluded by the sample-specific criteria due to different data qualities. The datasets for all samples were individually normalized by the comprehensive SCTransform function with default parameters to remove the differences in sequencing depth across cells, and the top 3000 highly variable genes were detected for each sample. All sample datasets were integrated with the identified anchors. The top 50 dimensions were obtained by performing principal component analysis (PCA) to get bidimensional coordinates for each cell, and the unsupervised clustering was conducted on the basis of shared nearest neighbor graphs to identify cell clusters. Moreover, the resolutions were set to 0.7 for the major cell types in liver, 0.5 for endothelial cells (ECs), 0.1 for HSCs, 0.1 for hepatocytes, 0.2 for KCs, and 0.05 for LCMs. The cell clusters were visualized by uniform manifold approximation and projection (UMAP), and the cell types were annotated on the basis of the prior knowledge of marker genes.

### Identification of DEGs and functional enrichment analysis

The identification of differential expressed genes (DEGs) for each cell type was implemented with “FindMarkers” or “FindAllMarkers” function in Seurat packages. The significant DEGs were detected by two parameters *P* < 0.05 and |log_2_FC|> 0.25. Gene Ontology (GO) analysis was performed by the R package clusterProfiler using the different types of significant DEGs. *P* < 0.05 was considered as significantly enriched for all the functional enrichment analysis.

### Immunofluorescence (IF) staining

Ten-micrometer liver frozen sections were acquired using Leica CM1860 and fixed with acetone/methanol (4:1) for 10 min at –20 °C. After blocking with 4% donkey serum (solarbio) in TBS for 1 h at room temperature, sections were incubated with primary antibodies (CD68, Desmin, or CX3CR1) diluted in TBS including 0.5% donkey serum overnight at 4 °C. Rat anti-mouse CD68 monoclonal antibody (#137002, 1:200 dilution) was from BioLegend, rabbit anti-Desmin polyclonal antibody (#16520-1-AP, 1:200 dilution) was from Proteintech, and rabbit anti-CX3CR1 polyclonal antibody (#2093, 20 μg/mL) was from Prosci. Next, appropriate Alexa Fluor-conjugated secondary antibodies against rat or rabbit (1:500 dilution) from Thermo Fisher Scientific were applied for 1 h at room temperature. Finally, the sections were mounted in Mowiol mounting medium, containing 12% Mowiol 4–88 (EMD Millipore), 30% glycerol, and 100 mM Tris pH 8.5, and imaged with a Leica TCS SP8 confocal microscope.

### Statistical analysis of IF-stained images

The area percentage of Desmin or CD68 and average intensity of CD68 were quantified using ImageJ (https://imagej.nih.gov/ij/). During the quantification, regions of interest (ROIs) were generated by the intensity segmentation of Desmin or CD68, and the area or intensity of each ROI was measured. The area percentage of Desmin was calculated as the ratio of the area of all the Desmin ROIs to the whole image area. The average intensity of CD68 was acquired by quantifying the ratio of the total intensity of all the CD68 ROIs to their total areas. The area percentage of CD68 was quantified as the ratio of the area of all the CD68 ROIs to the area denoted by Cx3cr1.

### Cell–cell communication

The R CellChat package was used to perform cell–cell communication analysis with default parameters based on the ligand–receptor interactions in different cell types. In brief, the normalized gene expression matrix and cell type meta-information from the control and GLY groups acted as input for generating CellChat objects. The two objects were merged for comparative analysis using the “mergeCellChat” function. The cell type labels were derived from 13 major cell types of liver tissues. The significant communication networks within all cell subsets were inferred by assigning each interaction with a probability value and performing a permutation test. At last, communication networks were visualized by chord diagram and bubble plot.

### Data availability

All the sequencing data have been deposited in Genome Sequence Archive (GSA) (https://bigd.big.ac.cn/gsa/) with the accession number of CRA007340.

## Results

### GLY induces liver damage in mice

**Fig. 1:**
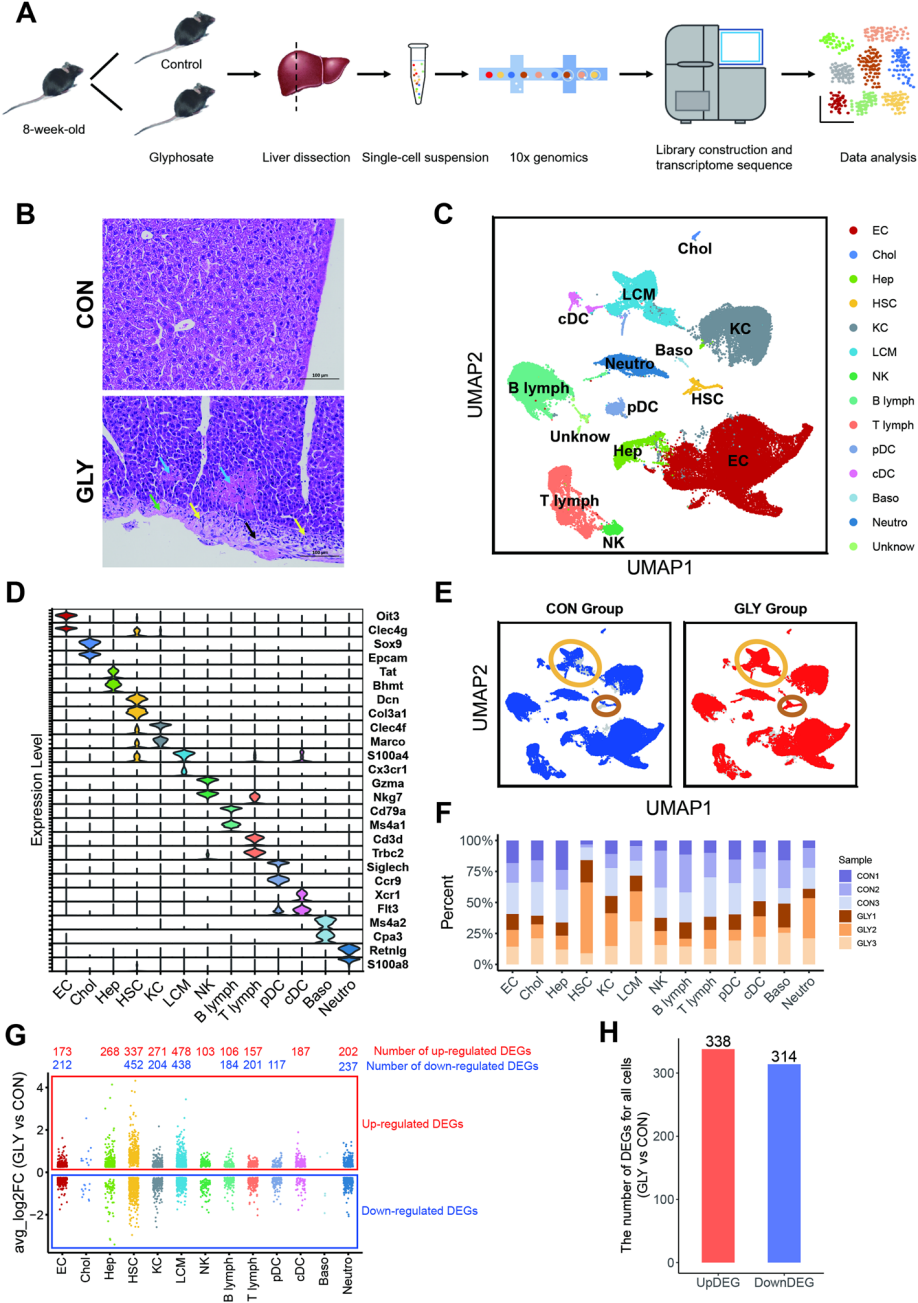
The global cell types and changes in mice liver tissues delineated by scRNA-seq. **A** An overview of the study design. **B** The H&E staining of liver tissue (scale bar, 100 μm). Note the fibroblast proliferation in the Glisson’s capsule (black arrow), exudation (green arrow), slight inflammatory cell infiltration (yellow arrow), and focal necrosis (blue arrow) in GLY-treated mice. **C** UMAP plot displaying 13 major cell clusters based on 60,348 single-cell transcriptomes. **D** Violin plots showing the expression of two well-established marker genes for each cell type. **E** Distribution comparison of clusters from control (CON) and GLY groups. **F** Percent contribution of control (blue) and GLY (orange) mouse liver cells for each cell type. Note that the relative percentages of HSCs and LCMs were significantly increased by GLY. **G** The distributions of upregulated and downregulated genes for each cell type after GLY treatment. Note that HSCs and LCMs contained the largest number of DEGs. **H** The number of upregulated and downregulated genes for all cells after GLY treatment. Note that HSCs and LCMs contained the largest number of DEGs

To investigate the potential hepatotoxicity of GLY at a single-cell level, C57BL/6 male mice were administered GLY for consecutive 7 days (Fig. 1A). Male mice were chosen since male animals suffered more acutely than females from liver damage [34]. Histopathological observation revealed fibroblast proliferation in the Glisson’s capsule (black arrow), exudation (green arrow), slight inflammatory cell infiltration (yellow arrow), and focal necrosis (blue arrow) in GLY-treated mice, suggesting that GLY can induce liver damage (Fig. 1B). Using the criteria in Additional file 1: Fig. S1A, the degree of necrosis of the liver tissue of each mouse from the CON- and GLY-treated group was examined. We found single-cell necrosis (numerical assessment number 1) in two of the three CON mice, while all three mice in GLY group showed more severe necrosis (numerical assessment numbers 3, 2, and 4) (Additional file 1: Fig. S1A). The reported pathological features of the liver after GLY exposure were cytoplasmic vacuolation, degeneration of hepatocytes, steatosis, multifocal necrosis, leukocyte infiltration, massive deposition of reticular fibers, and cytoplasmic glycogen deposition in hepatocytes [16, 34–36]. The histopathological effects of GLY on liver tissue in our study are in accordance with these studies using laboratory rats.

### Construction of mouse liver cell atlas upon GLY treatment

We performed droplet-based scRNA-seq (10x Genomics platform) of liver tissues from three control (CON) and three GLY-treated mice. After rigorous quality control, we obtained a total of 20,786 mouse genes from 60,348 qualified cells for subsequent analysis (Additional file 1: Fig. S1B). Of these, 27,733 cells (46%) were originated from GLY-treated samples and 32,615 cells (54%) were from control samples. The unsupervised clustering analysis partitioned all cells into 13 major cell lineages as displayed by uniform manifold approximation and projection (UMAP) plot (Fig. 1C and Additional file 1: Fig. S2A, B). The cell lineages were annotated by the expression of well-established markers, including endothelial cell (EC, expressing *Oit3* and *Clec4g*), cholangiocyte (Chol, expressing *Epcam* and *Sox9*), hepatocyte (Hep, expressing *Tat* and *Bhmt*), hepatic stellate cell (HSC, expressing *Dcn* and *Col3a1*), Kupffer cell (KC, expressing *Clec4f* and *Marco*), liver capsular macrophage (LCM, expressing *S100a4* and *Cx3cr1*), NK cell (NK, expressing *Gzma* and *Nkg7*), B lymphocyte (B lymph, expressing *Cd79a* and *Ms4a1*), T lymphocyte (T lymph, expressing *Cd3d* and *Trbc2*), plasmacytoid dendritic cell (pDC, expressing *Siglech* and *Ccr9*), conventional dendritic cell (cDC, expressing *Xcr1* and *Flt3*), basophil cell (Baso, expressing *Ms4a2* and *Cap3*), and neutrophil (Neutro, expressing *Retnlg* and *S100a8*) (Fig. 1D, Additional file 1: Fig. S2C and Additional file 2: Table S1).

Next, we quantified and compared the proportions of each cell type in control and GLY groups to reveal the effect of GLY treatment on cell composition. Overall, the healthy and GLY-treated mouse livers both contain the 13 cell clusters (Additional file 1: Fig. S2D). The relative percentages of HSCs and LCMs were significantly increased by GLY, whereas the proportions of ECs, hepatocytes, and lymphocytes were decreased compared with those in healthy liver tissues (Fig. 1E, F, Additional file 1: Fig. S2E and Additional file 2: Table S2). Transcriptomic analysis identified differentially expressed genes (DEGs) associated with GLY treatment in various cell types (Fig. 1G and Additional file 2: Table S3). Remarkably, HSCs and LCMs contained the largest number of DEGs. The number of DEGs from scRNA-seq analysis in HSCs and LCMs (Fig. 1G) is significantly higher than that of traditional analysis (Fig. 1H). These observations strongly suggest that GLY exerts hepatotoxicity effects by affecting specific cell (sub)types and corresponding genes.

### GLY exposure induces HSC activation and proliferation

**Fig. 2:**
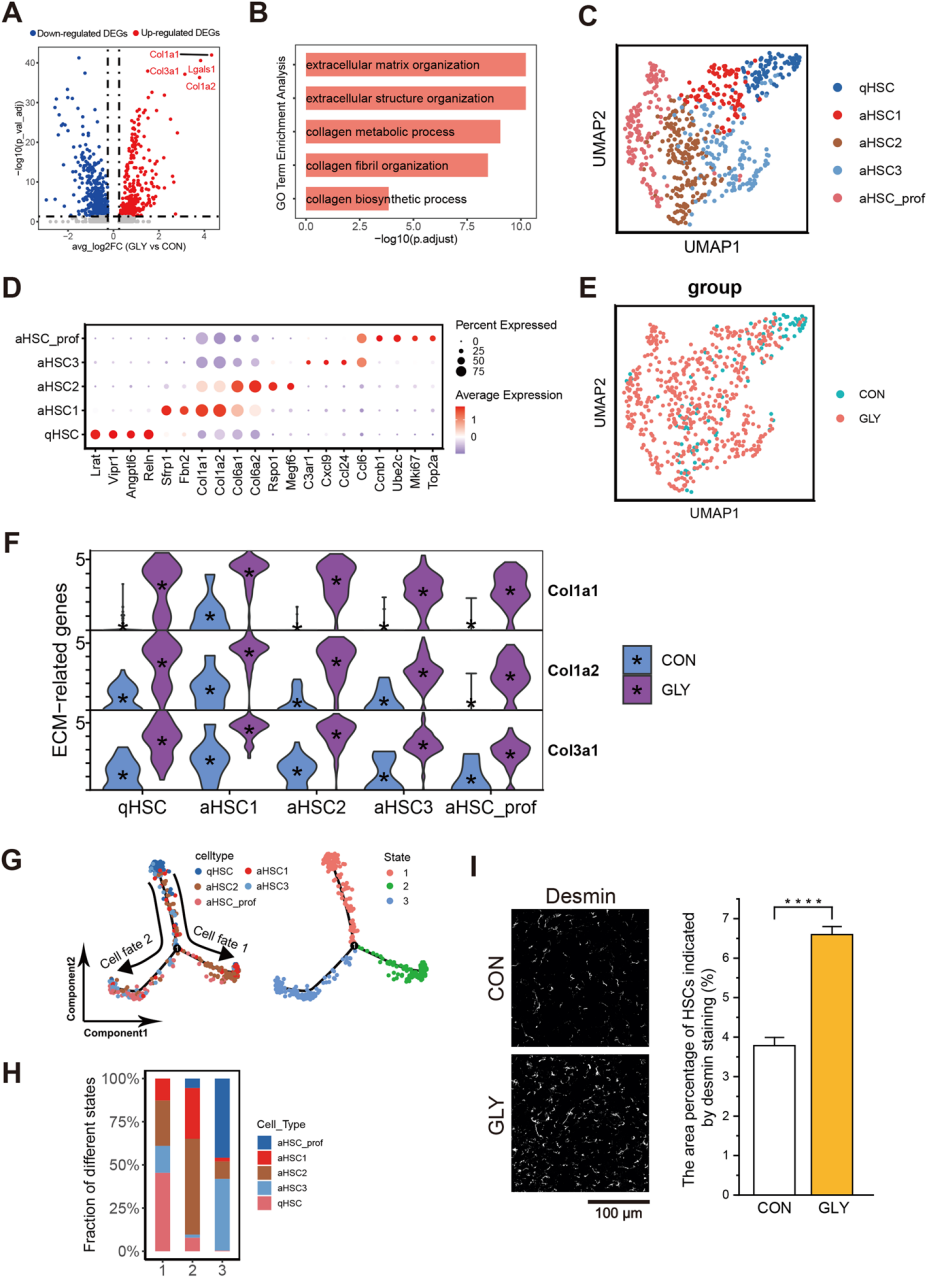
Transcriptomic changes of HSC activation after GLY stimulation. **A** Volcano plot of genes differentially expressed between control and GLY-treated HSCs. **B** GO analysis for upregulated DEGs of total HSCs after GLY treatment. **C** UMAP plot showing the cell subtypes of HSCs. **D** The expression of specific marker genes identified HSC subtypes. **E** Merged UMAP plot for control and GLY groups. **F** Violin plots of the expression for three genes associated with ECM. **G** Pseudotime trajectory analysis of HSC subtypes. **H** Proportion changes of each cell type for different states during the pseudotime analysis. **I** Typical images and bar graph showing the increased area percentage of Desmin after GLY treatment. Scale bar, 100 μm; Error bar, mean ± standard deviation (SD); *P* value is from Student’s *t* test (unpaired and two-tailed); ****P ≤ 0.0001; the quantification was based on six random areas from each group

HSCs reside in the subendothelial space of Disse, interposed between hepatocytes and LSECs. Upon liver injury, nonproliferative quiescent HSCs transdifferentiate into proliferative, inflammatory, and contractile myofibroblasts with enhanced extracellular matrix (ECM) production (also known as “cell activation”) [37]. GLY treatment dramatically increased the relative proportion and absolute numbers of HSCs (Figs. 1E, 2E and Additional file 1: Fig. S3A–C), along with upregulation of key genes involved in extracellular matrix organization, collagen metabolic process, collagen fibril organization, and collagen biosynthetic process (Fig. 2A, B and Additional file 2: Table S5, 6).

Clustering analysis of HSCs in both control and GLY mice identified five distinct subpopulations (Fig. 2C)—one quiescent subtype qHSC and four activated subtypes composed of aHSC1-3 and aHSC_prof—using the markers shown in Fig. 2D. The qHSC cluster accounted for the largest proportion (~ 44%) in control mice but dropped to less than 10% after GLY treatment, whereas the relative proportions of activated subtypes, particularly aHSC_prof and aHSC3, were increased by GLY treatment (Fig. 2E, Additional file 1: Fig. S3C and Additional file 2: Table S4). The aHSC_prof cluster was defined by the high expression of proliferation markers such as *Mki67* (Fig. 2D and Additional file 1: Fig. S3F), and aHSC3 was inflammation-related with strong expression of chemokines, including Ccl6, Ccl24, Cxcl9, and C3ar1 (Fig. 2D). DEG analysis identified that genes related to ECM (*Col1a1*, *Col1a2*, and *Col3a1*) were among the top ten upregulated ones after GLY exposure (Fig. 2F, and Additional file 2: Table S5). This finding suggested that GLY induces excessive deposition of fibrillary collagen, which may further activate HSCs and lead to liver fibrosis and other liver diseases [38].

To gain insight into the transdifferentiation fate of HSCs, pseudotime analysis was conducted to acquire the transcriptomic changes of HSCs during GLY exposure. We observed that the branch 1, primarily composed of quiescent HSCs from control group, developed into aHSC2-enriched branch 2 (cell fate 1) or aHSC3 and aHSC_prof-enriched branch 3 (cell fate 2), accompanied by other cell types (Fig. 2G, H and Additional file 1: Fig. S3D, E). Of note, nearly all the cells in branches 2 and 3 were activated HSCs from GLY-treated mice (Fig. S3D, E). The activation of HSCs is now well established as a key driver of fibrosis upon liver injury [39], and our data implied that GLY could induce HSC proliferation and activation. We next examined the expression of Desmin, a marker of HSC [40], in CON- and GLY-treated samples, and observed a significantly increased number of cells expressing Desmin (presented in area percentage, from 3.8% to 6.6%) upon GLY exposure which is consistent with our finding that GLY induced HSC proliferation (Figs. 1E, 2E, I).

Taken together, we observed activation of quiescent HSCs, including increase of proliferative and inflammatory HSCs, and enhancement of collagen genes, implying that GLY could induce liver damage.

### Dysregulation of hepatocyte signaling induced by GLY

**Fig. 3:**
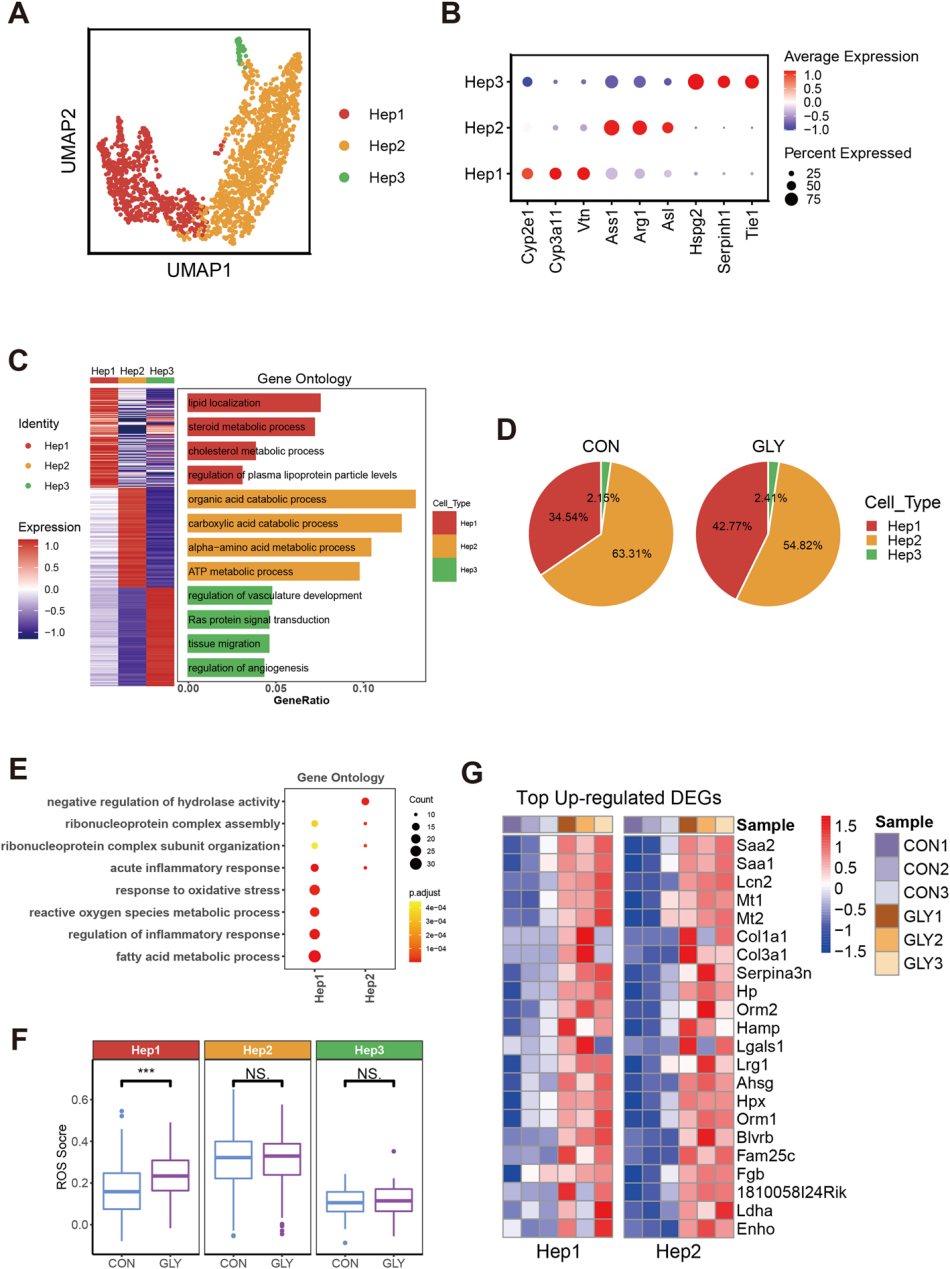
Specific changes of hepatocyte subsets between control and GLY groups. **A** UMAP plot for the three subtypes of hepatocytes termed Hep1, Hep2, and Hep3. **B** The expression of specific marker genes in hepatocyte subtypes. **C** Heatmap of DEGs for the three cell subtypes and their corresponding functional enrichment analysis. **D** Pie plots showing proportional changes of each subtype within the control and GLY groups. **E** GO analysis for upregulated DEGs of Hep1 and Hep2. **F** Boxplots of ROS score for each cell subtype. The middle lines indicate median values; boxes range from the 25th to 75th percentile. *P* value is from wilcox.test (unpaired and two-tailed); ***P < 0.001, NS, not significant. **G** Heatmaps of upregulated genes for Hep1 and Hep2 after GLY treatment

As GLY treatment reduced the proportion of hepatocytes (Fig. 1F and Additional file 1: Fig. S4A, B), we examined the subpopulations of this cell type. Hepatocytes were divided into three groups, namely Hep1, Hep2, and Hep3 (Fig. 3A, Additional file 1: Fig. S4C and Additional file 2: Table S7), using the specific genes listed in Fig. 3B. Functional enrichment revealed that different subtypes were highly involved in various processes (Fig. 3C and Additional file 2: Table S8). Hep1 was mainly involved in lipid metabolism such as lipid localization, steroid metabolic process, cholesterol metabolic process, and regulation of plasma lipoprotein particle levels. In Hep2, the enriched functions were focused on some catabolic processes such as organic acid catabolic process and carboxylic acid catabolic process. Hep3 participated in processes of vasculature development, tissue migration, and angiogenesis.

After GLY exposure, Hep1 displayed an increased proportion while a decreased trend was noted for Hep2 (Fig. 3D and Additional file 1: Fig. S4D). Hep3 accounted for only 2% of the total population, and no substantial difference in its composition was observed after GLY treatment (Fig. 3D and Additional file 1: Fig. S4D). Next, we examined the DEGs in the Hep1 and Hep2 (Fig. 3G and Additional file 2: Table S9). ROS metabolic process and ROS score were elevated in Hep1 of GLY-treated mice, which was the major contribution to the increased ROS score observed for all the hepatocytes (Fig. 3E, F and Additional file 1: Fig. S4E), suggesting that GLY induced oxidative stress in this subtype. A similar effect of GLY in causing oxidative stress has been reported in the SH-SY5Y cell line [41] and rat liver [17]. Mesnage et al. observed activated oxidative stress (reflected by Srxn1 and Blvrb) in mammalian stem cell-based ToxTracker system by glyphosate-based herbicides but not glyphosate itself [23], In our study, the expression level of Srxn1 and Blvrb (Fig. 3G and Additional file 2: Table S9) was improved after GLY exposure. The contradictory observation could be caused by different testing models (in vitro cell line versus in vivo animal model) or administered dose variance (≤ 5 mM versus 200 mg/kg). The dose-dependent toxicity of glyphosate has been reported by some researchers [16, 17, 42, 43]. One of the main events, induced by ROS and oxidative stress, is lipid peroxidation or even oxidative damage of lipids [44, 45]. Thus, we observed that the genes involved in fatty acid metabolic process were markedly upregulated in Hep1 (Fig. 3E and Additional file 2: Table S10). Besides, the expression of genes related to inflammatory response also increased after GLY treatment (Fig. 3E and Additional file 2: Table S10), displaying consistency with former statements that GLY could induce inflammatory response in multiple organs, including liver, lung, and small intestine [17, 46, 47]. In addition, upregulation of genes involved in ribonucleoprotein complex assembly and ribonucleoprotein complex subunit organization in both Hep1 and Hep2 suggests potential DNA damage of GLY (Fig. 3E), which has been reported previously [23]. Overall, GLY may cause oxidative stress, abnormal lipid metabolism, inflammatory response, and DNA damage, suggesting GLY-induced dysregulation of hepatocytes.

### Gene expression heterogeneity of KCs in GLY-treated mouse livers

**Fig. 4:**
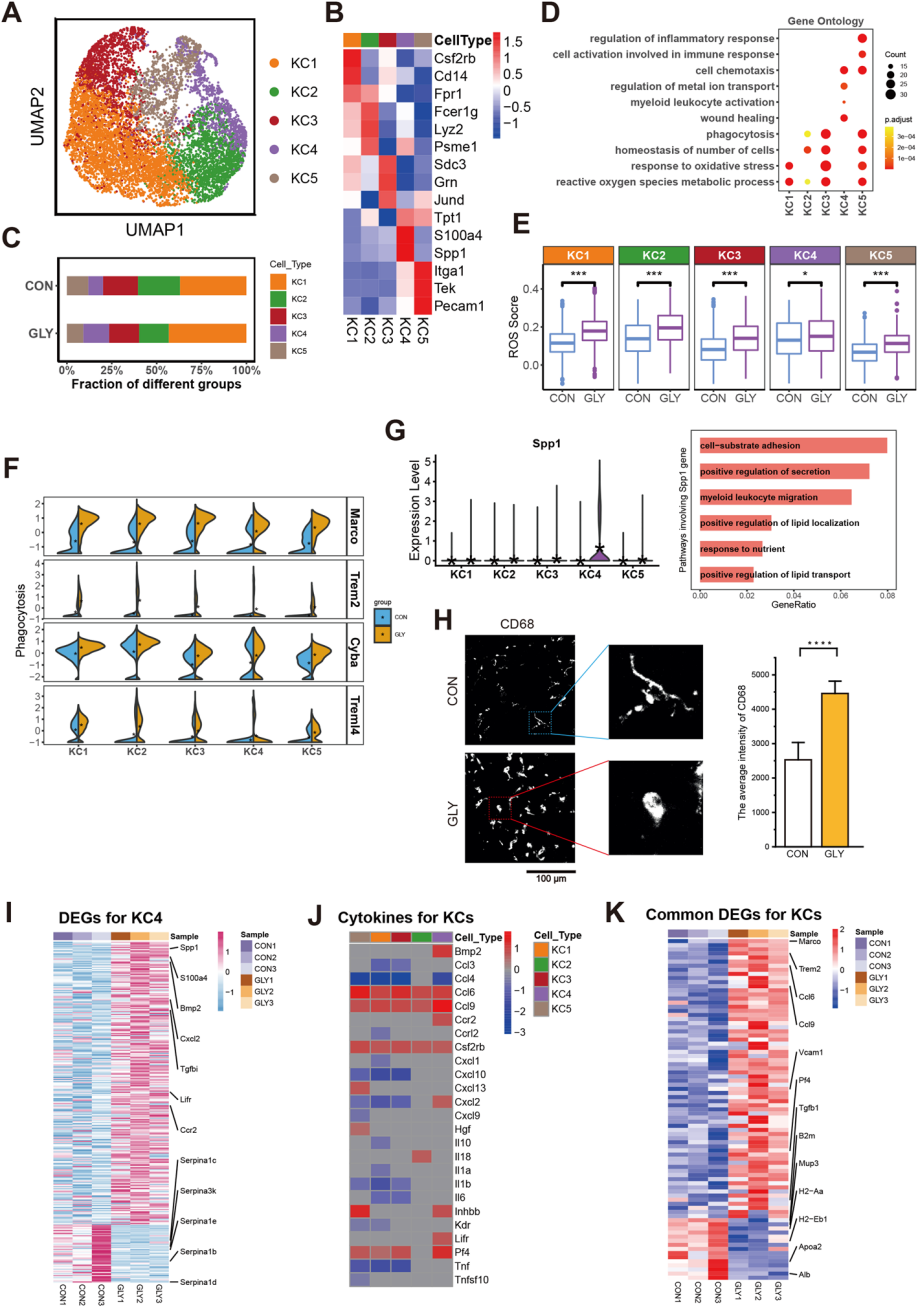
Transcriptomic changes of KCs after GLY treatment. **A** UMAP visualization of the five subtypes for KCs. **B** Heatmap of the expression levels of representative marker genes in KC subtypes. **C** Histogram showing proportional changes of each KC subtype for two groups. **D** GO analysis for upregulated DEGs of different cell subtypes. **E** Boxplots of ROS score for each cell subtype. The middle lines indicate median values, boxes range from the 25th to 75th percentile. *P* value is from wilcox.test (unpaired and two-tailed); ***P < 0.001, *P < 0.05. **F** Split violin plots of the expression of four genes associated with phagocytosis. **G** Violin plots comparing the expression level of *Spp1* in each subtype between control and GLY groups (left). GO analysis for upregulated DEGs of KC4 involving Spp1 gene (right). **H** Typical images and bar graph showing the increased expression level of CD68 after GLY treatment. Boxed regions are amplified on the right; scale bar, 100 μm; error bar, mean ± SD; *P* value is from Student’s *t*-test (unpaired and two-tailed); ****P ≤ 0.0001; the quantification was based on six random areas from each group. **I** Heatmap of upregulated and downregulated genes for KC4 in control and GLY samples. **J** Differentially expressed cytokines for each KC subtype after GLY treatment. **K** Heatmap for common upregulated and downregulated genes of all KC clusters

KCs, the resident macrophages of the liver, reside within the hepatic sinusoid together with other innate immune cells, such as natural killer cells, natural killer T cells and dendritic cells [48]. On the basis of the specific marker genes, KCs were further clustered into five subtypes: KC1 (expressing *Csf2rb*, *Cd14*, and *Fpr1*), KC2 (*Fcer1g*, *Lyz2*, and *Psme1*), KC3 (*Sdc3*, *Grn*, and *Jund*), KC4 (*Tpt1*, *S100a4*, and *Spp1*) and KC5 (*Itga1*, *Tek*, and *Pecam1*) (Fig. 4A, B and Additional file 1: Fig. S5A–C), and GLY treatment resulted in various proportional changes in these subtypes (Fig. 4C, Additional file 1: Fig. S5D, E and Additional file 2: Table S11). Next, we analyzed the GLY-induced DEGs in each subtype (Fig. 4D). KCs, especially KC1, KC3, and KC5, displayed enhancements in ROS level and oxidative stress response after GLY exposure (Fig. 4D, E and Additional file 1: Fig. S5G). We also noticed that genes associated with phagocytosis, such as *Macro* and *Cyba*, were upregulated in KCs (Fig. 4D, F and Additional file 1: Fig. S5F), suggesting that KCs might be activated by GLY to maintain the immunological homeostasis. It has been reported that the expression of CD68 (a macrophage marker) was enhanced after macrophage activation [49], and the increased CD68 expression after GLY treatment was also observed in our work (Fig. 4H). Since CD68 is a general macrophage marker, we checked its expression in the 13 major cell clusters identified in our study and noted that it was highly expressed in KCs, LCMs, pDCs, and cDCs (Additional file 1: Fig. S5H). The improved average intensity of CD68 should be more correlated with KCs since we excluded LCMs by quantifying non-liver capsular areas, and no significant difference of CD68 expression level in pDCs and cDCs was observed after GLY treatment and their numbers were much less than KCs. Besides, GLY induced some KCs polarized into a more circular morphology, an anti-inflammatory M2 state that is generally believed to promote tumorigenesis and tumor progression (Fig. 4H) [50, 51]. Collectively, these results further indicated that GLY might activate macrophages, including KCs.

By scrutinizing the DEGs in KCs, we noted that the GLY treatment remarkably increased the expression level of *Spp1* in subtype KC4 compared with the control group (Fig. 4G, I and Additional file 2: Table S12). *Spp1*, which encodes the secreted fibrogenic factor osteopontin (OPN), has been reported to be upregulated in liver fibrosis [52]. OPN has been found to activate HSCs [53–55] and be involved in some physiological cellular functions, including migration, adhesion, and secretion (Fig. 4G) [56, 57]. The expression of cytokines that were significantly affected by GLY is shown in Fig. 4J (Additional file 2: Table S13). Moreover, other liver-fibrosis-related genes, such as *Trem2* [58] and *Tgfbi* [59], also exhibited elevated expression levels (Fig. 4K and Additional file 2: Table S14). Thus, the results implied that GLY may potentially induce liver fibrosis.

### Activation of LCMs with GLY treatment

**Fig. 5:**
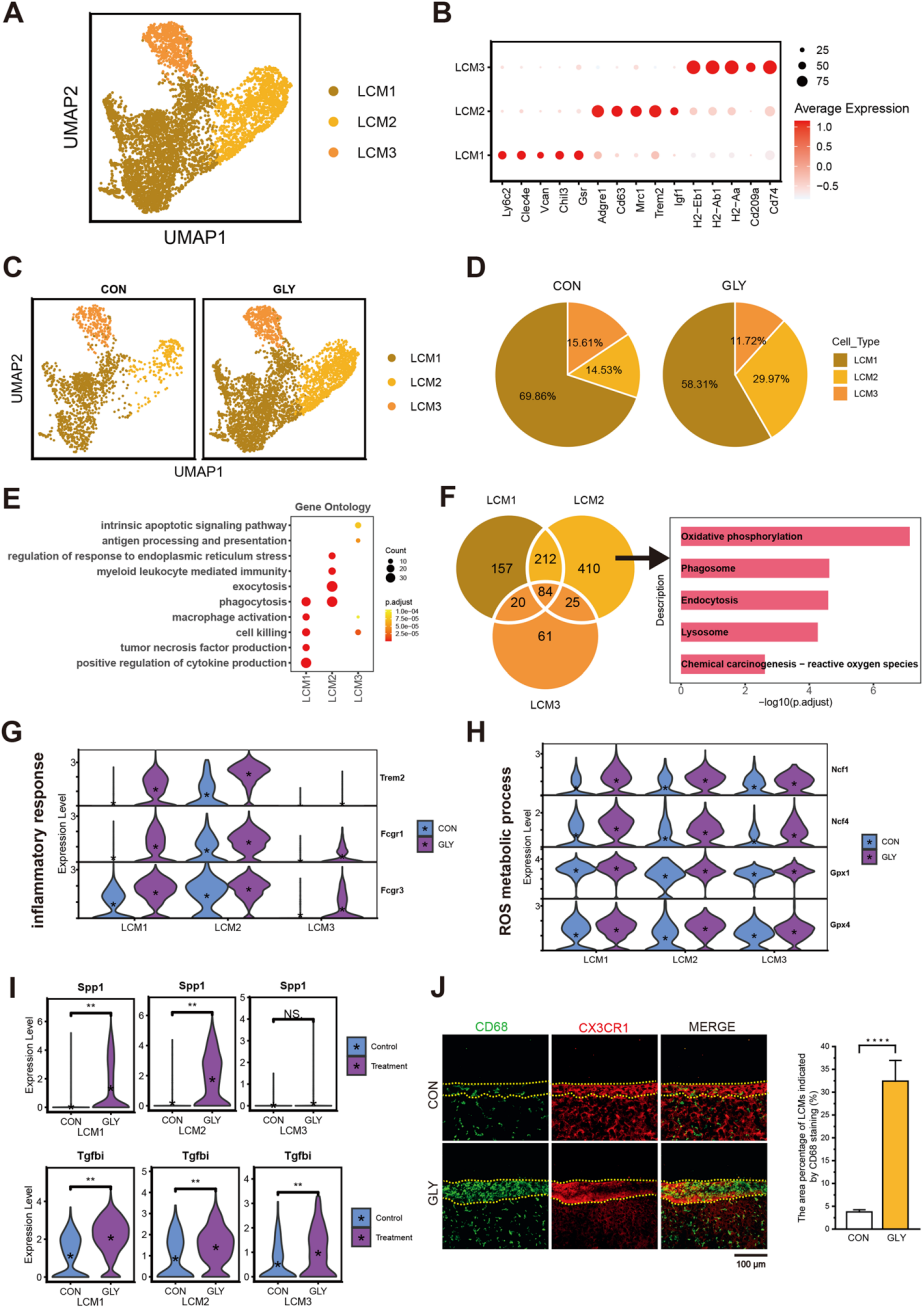
Inflammatory response and pro-fibrosis of LCMs after GLY treatment. **A** UMAP visualization of the three subtypes for LCMs. **B** The expression of specific marker genes identified the subtypes of LCM. **C** The distribution of each cell type in control and GLY groups. **D** Pie plots showing proportional changes of each cell type for two groups. **E** GO analysis for upregulated DEGs of different cell subtypes. **F** Venn diagram displaying the numbers of upregulated DEGs in the three subtypes (left). GO analysis for unique upregulated DEGs of LCM2 after GLY treatment (right). **G** Violin plots showing the expression levels of inflammatory response-related genes in each subtype. **H** Violin plots showing the expression levels of ROS metabolic process-related genes in each subtype. **I** Violin plots showing the expression levels of *Spp1* and *Tgfbi* genes in each subtype. *P* value is from wilcox.test (unpaired and two-tailed); **P < 0.001; NS, not significant. **J** Typical images and bar graph showing the increased area percentage of CD68 in the liver capsule after GLY treatment. The liver capsule outlined by yellow-dotted lines was denoted by Cx3cr1 staining; scale bar, 100 μm; error bar, mean ± SD; *P* value is from Student’s *t*-test (unpaired and two-tailed); ****P ≤ 0.0001; the quantification was based on eight random areas from each group

Three LCM subsets were revealed by unique transcriptomic signatures and visualized in UMAP plot, termed LCM1, LCM2, and LCM3 (Fig. 5A and Additional file 1: Fig. S6A, B). We annotated the subclusters with multiple DEGs and analyzed functional enrichment of these genes (Fig. 5B and Additional file 1: Fig. S6D). For instance, highly expressed genes in LCM1, including *Ly6c2*, *Clec4e*, and *Vcan*, were involved in positive regulation of cytokine production, regulation of inflammatory response, and phagocytosis. LCM2 strongly expressed *Adgre1*, *Cd63*, *Mrc1*, and *Trem2*, genes involved in cell chemotaxis, macrophage migration, and activation. Antigen processing and presentation genes (*H2-Eb1*, *H2-Ab1*, and *H2-Aa*) were prominently expressed by LCM3. Overall, the number of LCMs was significantly increased after GLY treatment (Fig. 5C and Additional file 2: Table S15). After GLY exposure, LCM2 was the most increased cluster among the three, as shown by either absolute number or relative percentage of cells (Fig. 5D and Additional file 1: Fig. S6C). The increased number of LCMs after GLY treatment was confirmed by CD68 staining (Fig. 5J). Since the LCMs are located in the hepatic capsule, we co-stained the LCM marker Cx3cr1 with CD68 to differentiate LCMs from other macrophages and found that the number of LCMs (displayed by area percentage) was significantly increased from 3.8% to 32.4% upon GLY exposure (Fig. 5J). GLY treatment induced upregulation of unique genes tightly related to oxidative phosphorylation, phagosome, endocytosis, lysosome, and ROS in LCM2 (Fig. 5F and Additional file 1: Fig. S6E). There are also several genes and pathways commonly dysregulated after GLY treatment across different subclusters (Fig. 5E and Additional file 2: Table S16). Notably, LCM1 and LCM2 showed increased expression of genes (*Trem2*, *Fcgr1*, and *Fcgr3*) related to inflammatory response in GLY group (Fig. 5G and Additional file 1: Fig. S6G). The expression levels of ROS metabolic process genes, including *Ncf1*, *Ncf4*, *Gpx1*, and *Gpx4*, were enhanced by GLY in all subclusters (Fig. 5H and Additional file 1: Fig. S6H). More importantly, GLY treatment induced the high expression of *Spp1*, which was closely associated with liver fibrosis and nonalcoholic steatohepatitis (NASH) (Fig. 5I and Additional file 1: Fig. S6F). *Tgfbi*, the gene encoding a potent profibrotic cytokine, was also overexpressed after GLY treatment among LCM subclusters (Fig. 5I). Meanwhile, the expression of several chemotactic cytokines was significantly increased by GLY in all LCM subsets (Additional file 1: Fig. S6I).

### Intercellular communication in crucial subtypes

The above findings have demonstrated that certain cell types in liver tissue underwent significant proportional and transcriptional alterations following GLY treatment, particularly LCMs, KCs, and HSCs. These aberrations may induce massive and complex changes of cell–cell communication networks in response to GLY stimulation. To disclose the underlying signaling patterns between cells, the statistical and biological communication networks were constructed for significant ligand–receptor pairs using CellChat program (Additional file 1: Fig. S7A). By comparing the detailed signaling features of the two groups, we identified that GLY treatment caused increased counts of interaction, as well as enhancement of interaction strength (Additional file 1: Figs. S7A, 8A). It is prominent that the interaction strength between LCMs and KCs increased significantly in the GLY group (Additional file 1: Figs. S7B, 8B). Our transcriptional analysis of LCMs has found that GLY increased the expression of *Spp1* (Additional file 1: Fig. S6I), which could induce the activation and infiltration of macrophages and generate pro-inflammatory environment through binding to integrin [60, 61]. Further ligand–receptor pair analysis on this pathway demonstrated that LCMs are the source of SPP1 ligands that act on LCMs, DCs, and KCs after GLY exposure (Additional file 1: Figs. S7C, 8F). Particularly, the interplays between SPP1 and integrin were captured only in the GLY group, but not in the control group (Additional file 1: Figs. S7D, 8D). In addition, we also identified some unique signaling pathways in either GLY or control group. For instance, PTN, FGF, and ncWNT, the signaling pathways involved in injury repair and liver metabolism [62–64], were present only in the GLY group, while BMP10 signaling pathway was present only in control (Additional file 1: Figs. S7E, 8C, E). Notably, HSCs acted as source cells for all these unique pathways, further highlighting the importance of HSCs in initiating GLY-induced liver damage. We also analyzed the upregulated and downregulated ligand–receptor pairs caused by GLY treatment (Additional file 1: Fig. S7F). On one hand, the upregulated ligands (C3, Ccl6, Ccl9, and Spp1) mainly came from cholangiocytes, hepatocytes, LCMs, and KCs, and the corresponding receptors (ITGAM_ITGB2, C3ar1, Ccr1, and ITGAV_ITGB5) existed mainly in LCMs. On the other hand, the downregulated ligands (Vegfa, Cxcl12, Sema3d, and Tnf) came mainly from cholangiocytes, HSCs, LCMs, and KCs, and these downregulated ligands acted on receptors of HSCs, LCMs, and hepatocytes (FLT1_KDR, Kdr, NRP2, PLXNA4, Cxcr4, and Tnfrsf1b).

## Discussion

Previous studies demonstrated that GLY might induce liver damage and dysregulated lipid metabolism in rats and mice. These studies accessed the hepatotoxicity by liver function test that measures alanine aminotransferase, aspartate aminotransferase, lactate dehydrogenase, and serum lipoproteins [16, 17], or by chemical biology that identified GLY targeting proteins [18]. Webster et al. performed transcriptomic profiling on livers of juvenile female brown trout and showed generation of oxidative stress and induction of cellular stress response pathways after GLY exposure [65]. Mesnage et al. conducted a transcriptome microarray analysis of GLY-based herbicide Roundup treatment of rat liver and kidney indicating damaged liver and kidney with a set of genes with altered expression level [66]. Jia et al. reported expression level changes of genes involved in ion transport, lipid metabolism, and peroxisome proliferator-activated receptor signaling pathway in livers of tilapia by transcriptomic analysis [67]. Another study from Mesnage et al. utilized multiple omics including transcriptomics showing that GLY-based herbicide MON 52276 increased hepatic steatosis and necrosis and both MON 52276 and glyphosate induced oxidative damage to DNA in rat livers [23]. Although providing valuable information in reflecting the toxic effects of GLY on the liver, these studies explored the transcriptomic changes as a whole without differentiating specific cell types. The widespread usage of GLY poses potential risks to the health of humans and animals and induces environmental contamination. Therefore, revealing the molecular mechanisms of the GLY-imposed toxicity could help us find solutions to relieve or resolve these concerns. Our study provides the first comprehensive single-cell transcriptional landscape of livers from healthy and GLY-treated mice. scRNA-seq technology can help to locate the specific cell (sub)types that were affected by GLY treatment. We demonstrated that GLY exerts hepatotoxicity by affecting specific cell (sub)types and corresponding genes. Our findings may provide a theoretical basis for assessing the potential hazards of GLY-based pesticides and help to explore the etiology of their hepatotoxicity.

### GLY leads to enhanced ROS production, oxidative stress, and inflammatory response in several cell types

ROS are products of normal cellular metabolism and are composed of superoxide anion (O_2_^−^), hydroxyl radical (OH^−^), hydrogen peroxide (H_2_O_2_), nitric oxide (NO), and singlet oxygen (^1^O_2_) [68]. The imbalanced regulation of ROS as well as dysregulated antioxidant factors result in oxidative stress during stress and injury [69], whereas continued oxidative stress would be developed into chronic inflammation [70]. In addition, ROS work as a vital mediator of angiogenesis, which is a key event in the progression of liver diseases, such as nonalcoholic fatty liver disease (NAFLD), NASH, and liver cirrhosis [71–74]. Multiple researchers have shown that oxidative stress, inflammatory response, and lipid metabolism disorder were the most common pathogenic features of GLY-induced liver injury. The overproduction of ROS could alter the levels and activities of antioxidants including superoxide dismutase (SOD), catalase (CAT), and glutathione peroxidase (GPx) [75–77]. Oxidative stress can activate inflammatory reaction pathways leading to the production of proinflammatory factors (*IL-1β*, *IL-6*, *IL-10*, *IFN-γ*, *TNF-α*) [47, 76, 78, 79]. In addition, ROS and oxidative stress may result in lipid peroxidation or even oxidative damage of lipids [44, 45]. In our study, we found that the GLY exposure induced oxidative stress, inflammatory response, and abnormal lipid metabolism in hepatocytes (Fig. 3E, F). Moreover, signaling pathways related to ROS metabolic process and regulation of inflammatory response were affected by GLY in KCs and LCMs (Figs. 4D, E, 5G, H and Additional file 1: Fig. S5G, 6G, H). Overall, our findings showed that GLY could induce hepatotoxicity by excessive ROS production, oxidative stress, and inflammatory response in certain cell populations. Liu et al. demonstrated GLY-induced hepatic damage started from oxidative stress, followed by inflammatory response, and finally lipid metabolism disorder. These processes were intimately interrelated with each other during GLY exposure [80]. Combining our study, the possible mechanism for the toxicity of GLY in hepatocytes, KCs, and LCMs is demonstrated below. GLY exposure caused overproduction of ROS, which resulted in the imbalance between ROS and antioxidant factors. Then the imbalance results in oxidative stress, followed by activation of inflammatory reaction pathways to produce proinflammatory factors such as *IL-1β*, *IL-6*, *IL-10*, *IFN-γ*, and *TNF-α* to introduce inflammatory response in the liver. Furthermore, oxidative stress and inflammatory response may result in abnormal lipid metabolism.

### GLY induces the activation of macrophages

KCs, the embryonically derived liver-resident macrophages, are highly phagocytic and self-renewing. They engulf pathogens from the portal or arterial circulation, gut-derived immunoreactive materials, and dead erythrocytes or cells from the systemic circulation, constituting the major phagocytic activity of the mononuclear phagocytic system [81]. LCMs are another type of liver macrophage derived from bone marrow and replenished from circulating monocytes [82]. LCMs are located in the hepatic capsule and protect the peritoneal cavity against pathogens [82]. Our data showed that genes associated with phagocytosis, such as *Macro* and *Cyba*, were upregulated in KCs (Fig. 4D, F and Additional file 1: Fig. S5F). The number of LCMs was significantly increased after GLY treatment (Fig. 5C, J and Additional file 2: Table S15). In the subcluster LCM2, GLY treatment induced upregulation of unique genes tightly related to phagosome, endocytosis, and lysosome (Fig. 5F and Additional file 1: Fig. S6E). Collectively, these results implied that GLY treatment might induce liver damage and thus activate macrophages to maintain the immunological homeostasis.

### GLY-exposed mouse livers display characteristics of liver fibrosis

HSCs, most of which were under nonproliferative and quiescent state in control mice, transdifferentiated into proliferative, inflammatory, and contractile myofibroblasts upon GLY exposure. DEG analysis identified that genes related to ECM (*Col1a1*, *Col1a2*, and *Col3a1*) were among the top ten upregulated ones after GLY exposure in HSCs (Fig. 2F and Additional file 2: Table S5). This finding suggested that GLY induces excessive deposition of fibrillary collagen, which may further activate HSC and lead to liver fibrosis and other liver diseases [38]. GLY treatment induced the high expression of *Spp1* gene, which was closely associated with liver fibrosis and NASH in LCMs (Fig. 5I and Additional file 1: Fig. S6F). Compared with control, *Tgfbi*, the gene encoding a potent profibrotic cytokine, was also overexpressed after GLY exposure among multiple LCM subclusters (Fig. 5I). For KCs, the fibrosis-related genes (*Spp1*, *Trem2*, and *Tgfbi*) were also observed to be upregulated (Fig. 4H, J and Additional file 2: Table S12). Thus, the results indicated that GLY may potentially induce liver fibrosis.

## Conclusion

Taken together, the present study provides a comprehensive single-cell transcriptional picture of the hepatotoxicity after GLY exposure. GLY induced changes in cell composition, transcription, and cell–cell communication, and exerted hepatotoxicity effects by affecting specific cell (sub)types and corresponding genes. GLY enhances ROS production, oxidative stress, and inflammatory response in multiple cell populations, and activates HCSs and macrophages to potentially induce liver injury. Although our work offers novel insights into the molecular mechanisms of the GLY-associated hepatotoxicity, we have to admit that one limitation of our study is the use of a much higher dose of GLY than common levels existing in the air, water, food, etc. However, it is reasonable to administer animals with maximum tolerated doses for toxicity test. Therefore, our study is still meaningful in revealing the potential molecular mechanisms of the hepatotoxicity effects of GLY. Of course, deconstructing the transcriptomes of the liver at the single-cell level by scRNA-seq under public exposed GLY level merits further investigation.

## Supplementary Information

**Additional file 1: Fig. S1. **Quality control for scRNA-seq. (A) Criteria for the degree of necrosis (left). Histopathological evaluation for all samples (right). (B) Consistency (nFeature_RNA, nCount_RNA, percent.mt) of cell capture and identification for each sample (left) and the two groups (right) after scRNA-seq data quality control. nFeature_RNA, nCount_RNA and percent.mt represent the number of genes, the number of transcripts and the percentage of mitochondrial genes of each cell, respectively. **Fig. S2. **Single-cell analysis of 13 major cell lineages. (A) UMAP showing all clusters from the control and GLY-treated groups. (B) Merged UMAP plot for all samples. (C) Heatmap of top 100 DEGs for major cell types. (D) Distribution comparison of the major cell clusters between the control and GLY-treated groups. (E) Histograms depicting the proportional changes of each cell type for individual samples (left) and the combined control and GLY-treated samples (right). **Fig. S3. **Single-cell analysis of HSCs. (A) UMAP showing all subclusters of HSCs from the control and GLY-treated groups. (B) Merged UMAP plot for all samples. (C) Proportional changes of each cell subtype for the two groups. (D) Pseudotime trajectory analysis implying the development of HSC subtypes. (E) Histogram displaying the proportional changes of the two groups under different states during the pseudotime analysis. (F) Violin plots showing the cell-cycle-related genes of each HSC subtypes. **Fig. S4. **Single-cell analysis of hepatocytes. (A) UMAP showing hepatocytes composed of three subclusters from the control and GLY-treated groups. (B) Merged UMAP plot for hepatocytes of all samples. (C) UMAP depicting the distribution of hepatocyte subclusters from the control and GLY-treated groups. (D) Histogram displaying the proportional changes of each subtype for every sample. (E) Boxplot of ROS score for the two groups. *P* value is from wilcox.test (unpaired and two-tailed); *P < 0.05. **Fig. S5. **Single-cell analysis of KCs. (A) UMAP showing all subclusters of KCs from the control and GLY-treated groups. (B) Dotplot showing the expression of representative markers in each KC subcluster. (C) Merged UMAP plot for all samples. (D) Histogram demonstrating the proportional changes of each KC subcluster for each sample. (E) UMAP displaying the distribution of each KC subcluster from the control and GLY-treated groups. (F) Boxplot of phagocytosis score for the two groups. *P* value is from wilcox.test (unpaired and two-tailed); ***P < 0.001, **P < 0.01, *P < 0.05. (G) Boxplot of ROS score for the two groups. *P* value is from wilcox.test (unpaired and two-tailed); ***P < 0.001. (H) Violin plots showing the expression levels of Cd68 genes for two groups in all cell clusters. **Fig. S6. **Single-cell analysis of LCMs. (A) UMAP showing the cell subclusters of LCMs from the control and GLY-treated groups. (B) Merged UMAP plot for all the samples. (C) Histogram visualizing the proportional changes of each LCM subtype for each sample. (D) Heatmap of all DEGs for the three LCM subtypes (left) and their corresponding functional enrichment analysis (right). (E) Venn diagram for the downregulated genes of each LCM subcluster after GLY treatment. (F) UMAP showing the expression level of *Spp1* in LCM subtypes of the two groups. (G) Boxplot of inflammatory response score for the two groups. (H) Boxplot of ROS score for the two groups. *P* value is from wilcox.test (unpaired and two-tailed); **P < 0.01. (I) Heatmap displaying the differentially expressed cytokines in each LCM subtype after GLY treatment. **Fig. S7.** Cell–cell communication analysis for major cell clusters. (A) The overall networks of cell–cell communication within different cell types for control and GLY groups. (B) The interaction strength of each cell type for control and GLY groups. (C) The change for SPP1 signaling pathway network after GLY treatment. (D) Detailed ligand–receptor interactions of the SPP1 signaling pathway for LCM cells. (E) Signaling pathways presented only in the GLY group. (F) Upregulated and downregulated signaling after GLY treatment. **Fig. S8.** Cell–cell communication analysis. (A) Histogram showing the numbers of inferred interactions and interaction strength of the control and GLY-treated groups. (B) Chord diagram exhibiting the numbers of inferred interactions of LCMs and KCs for the two groups. (C) Histogram displaying the relative information flow of the two groups. (D) Dotplot depicting the ligand–receptor pairs between LCMs and major cell lineages. (E) Chord diagram demonstrating the signaling pathways present only in the control group. (F) Violin plots showing the expression levels of ligand–receptor genes of the Spp1 signaling pathway for major cell lineages.

**Additional file 2: Table S1.** The top 100 genes for 13 major cell lineages. **Table S2.** The cell number for 13 major cell lineages. **Table S3.** The differentially expressed genes for 13 major cell lineages after glyphosate treatment. **Table S4.** The cell number for HSC cell subtypes. **Table S5.** The differentially expressed genes for HSC cell after glyphosate treatment. **Table S6.** The GO analysis for upregulated DEGs of HSC cells after GLY treatment. **Table S7.** The cell number for hepatocyte cell subtypes. **Table S8.** The differentially expressed genes for hepatocyte cell subtypes. **Table S9.** The differentially expressed genes for hepatocyte cell subtypes after GLY treatment. **Table S10.** The GO analysis for upregulated DEGs of hepatocyte cells after GLY treatment. **Table S11.** The cell number for Kupffer cell subtypes. **Table S12.** The average expression level of differentially expressed genes of KC4 cell for each sample. **Table S13.** The average fold change (avg_logFC) for differentially expressed cytokines of each Kupffer cell subtype after GLY treatment. **Table S14.** The average expression level of common differentially expressed genes of KC cell subtypes for each sample. **Table S15.** The cell number for LCM cell subtypes. **Table S16.** The differentially expressed genes for LCM cell subtypes after glyphosate treatment.

## Data Availability

All the sequencing data have been deposited in Genome Sequence Archive (GSA) (https://bigd.big.ac.cn/gsa/) with the accession number CRA007340.

## Abbreviations

GLY: Glyphosate
scRNA-seq: Single-cell RNA sequencing
KCs: Kupffer cells
HSCs: Hepatic stellate cells
LCMs: Liver capsular macrophages
DEGs: Differentially expressed genes
GO: Gene Ontology
UMAP: Uniform manifold approximation and projection
ECM: Extracellular matrix
