# Supplementary figures and images for: Single-cell transcriptome analysis reveals liver injury induced by glyphosate in mice
Source: Cell Mol Biol Lett. 2023 Feb 4;28:11. doi: 10.1186/s11658-023-00426-z (PMC9898913; doi:10.1186/s11658-023-00426-z)

**Fig. S1**


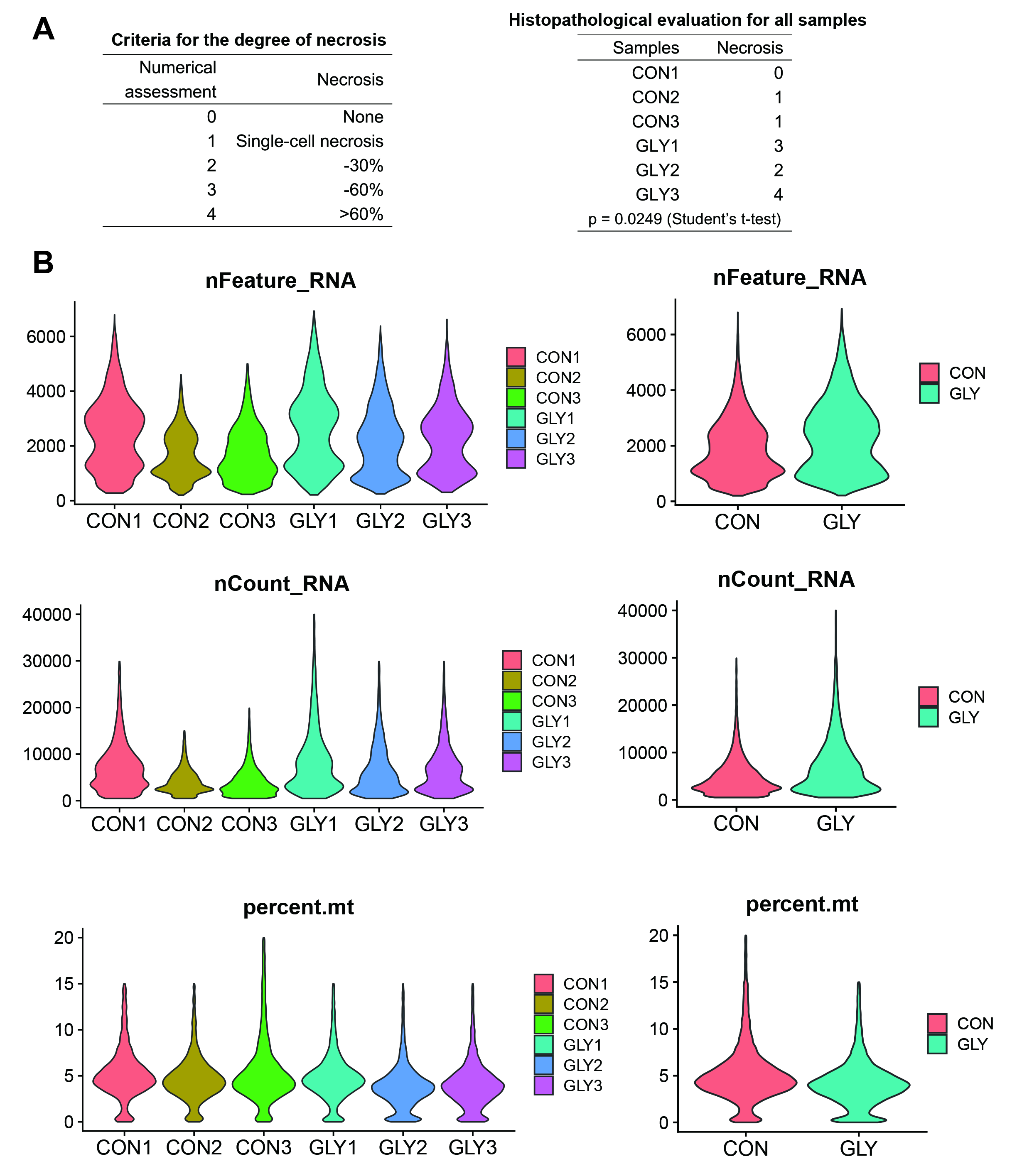


**Fig. S2**


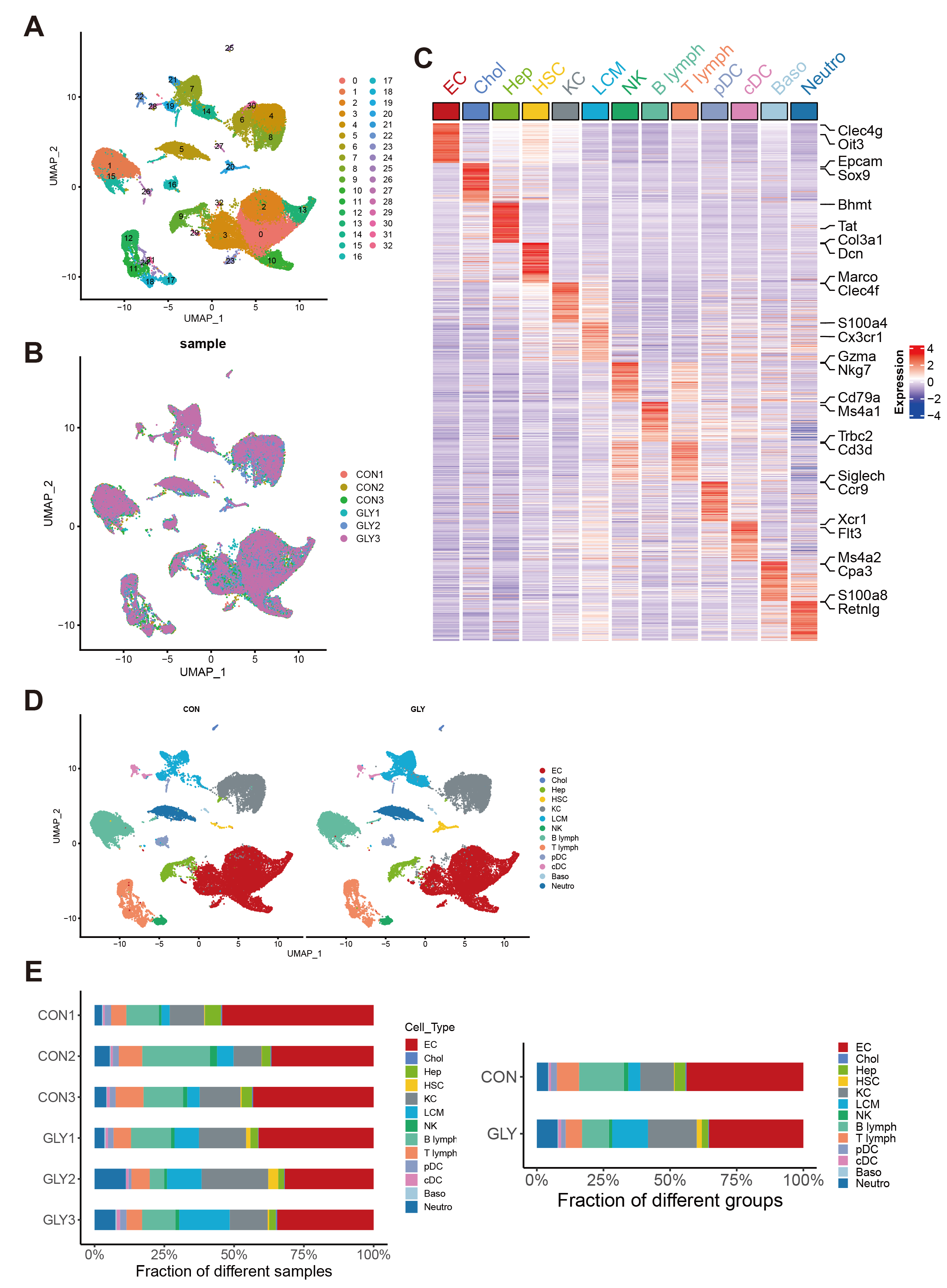


**Fig. S3**


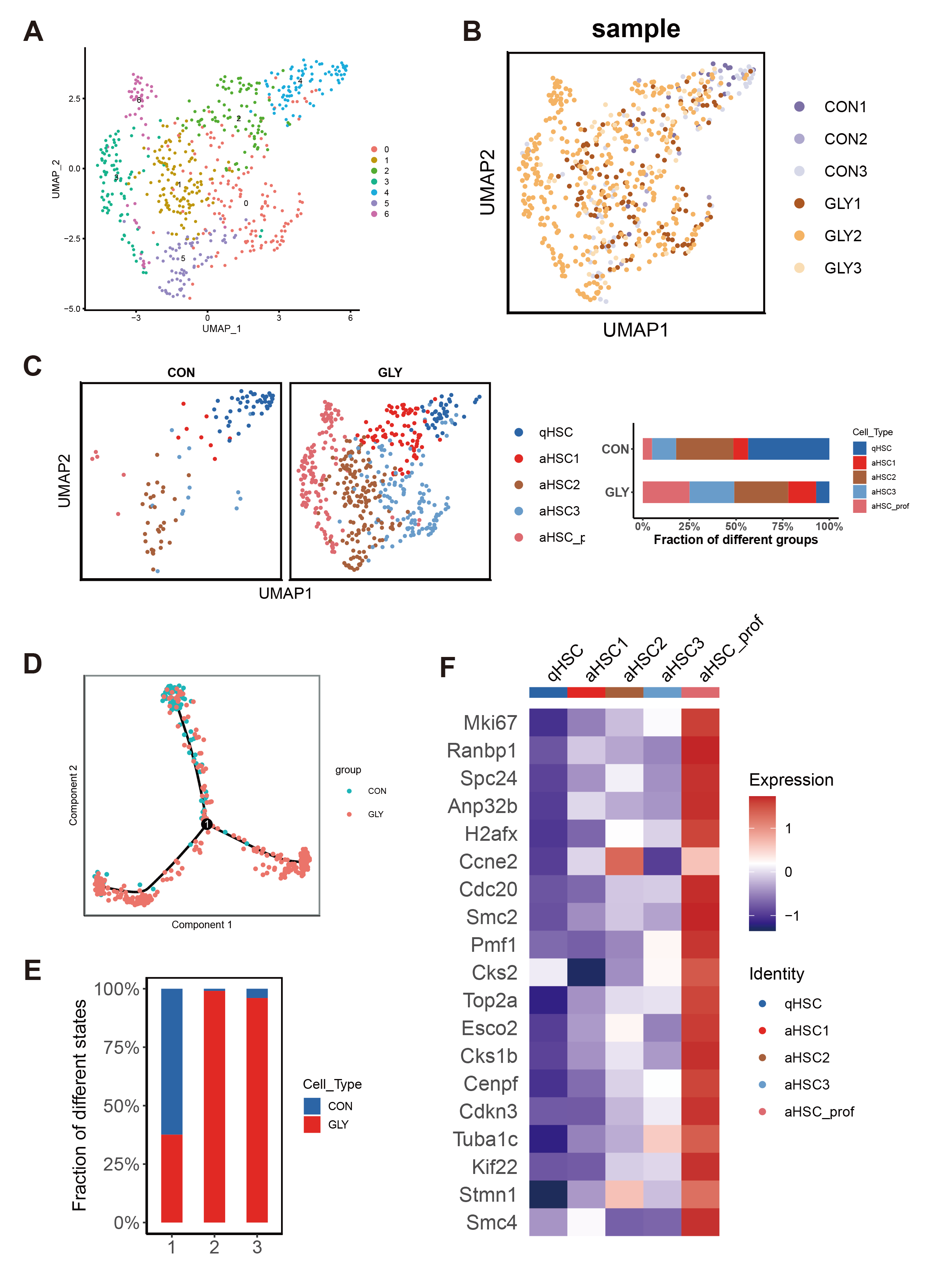


**Fig. S4
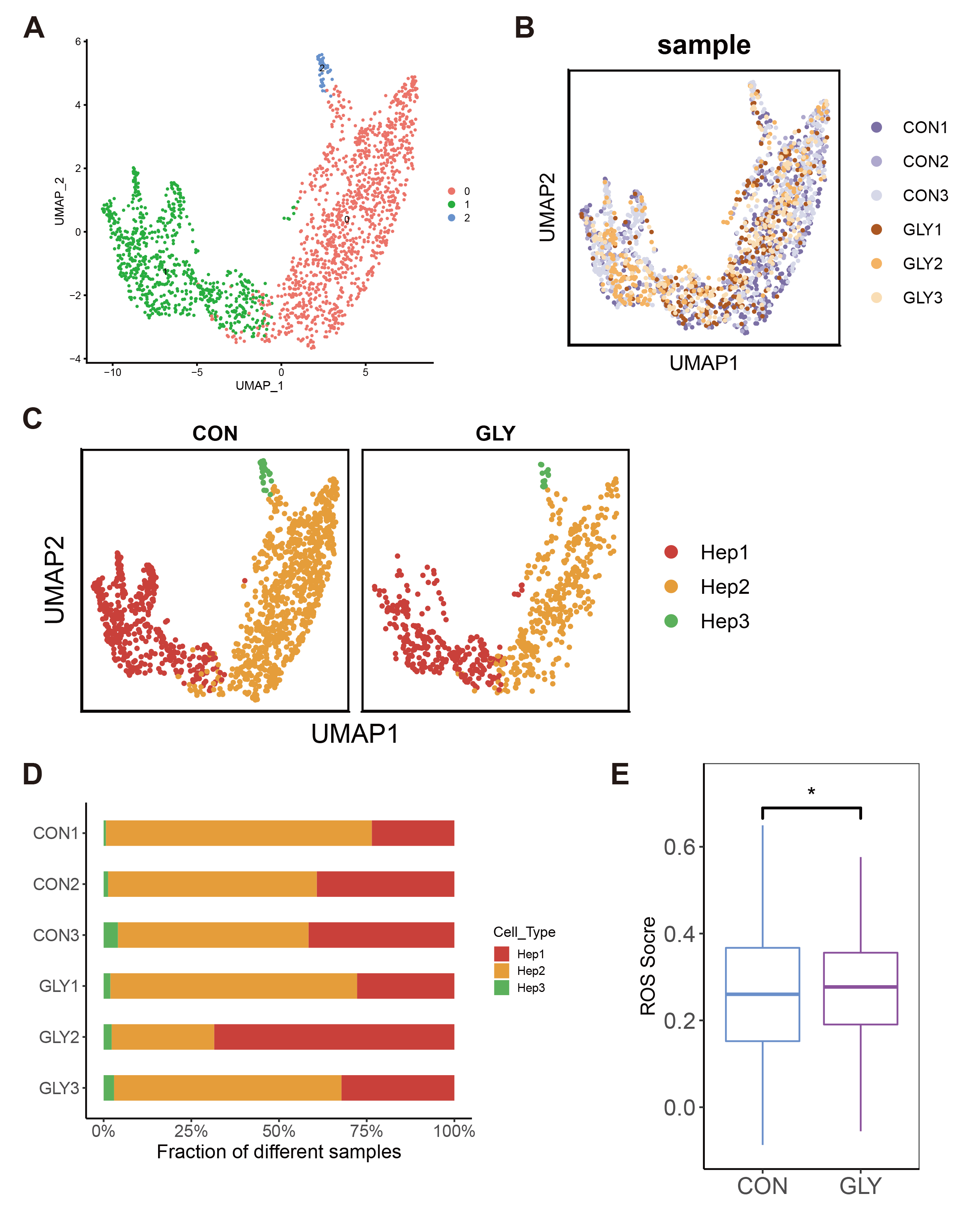
**

**Fig. S5
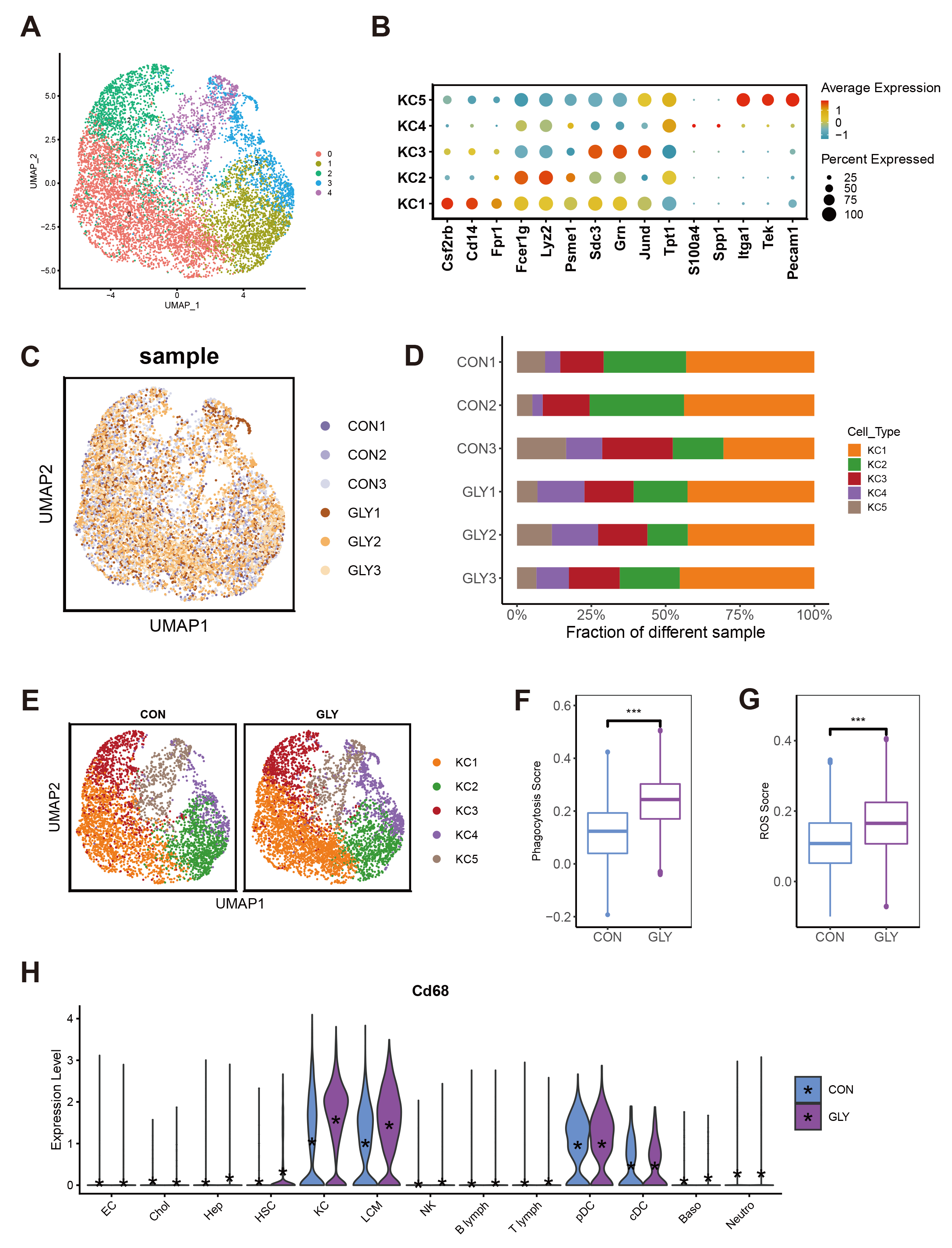
**

**Fig. S6**


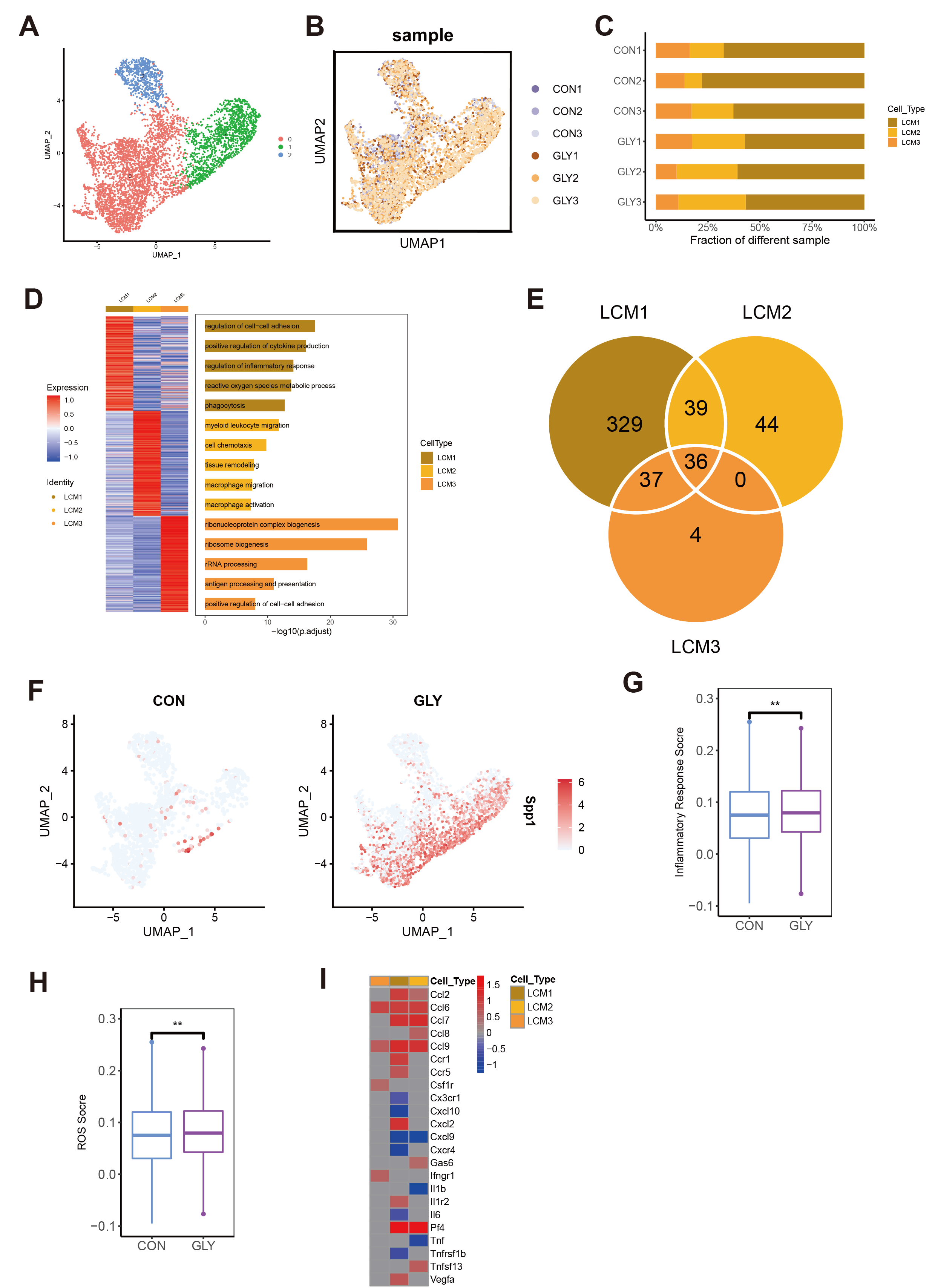


**Fig. S7**


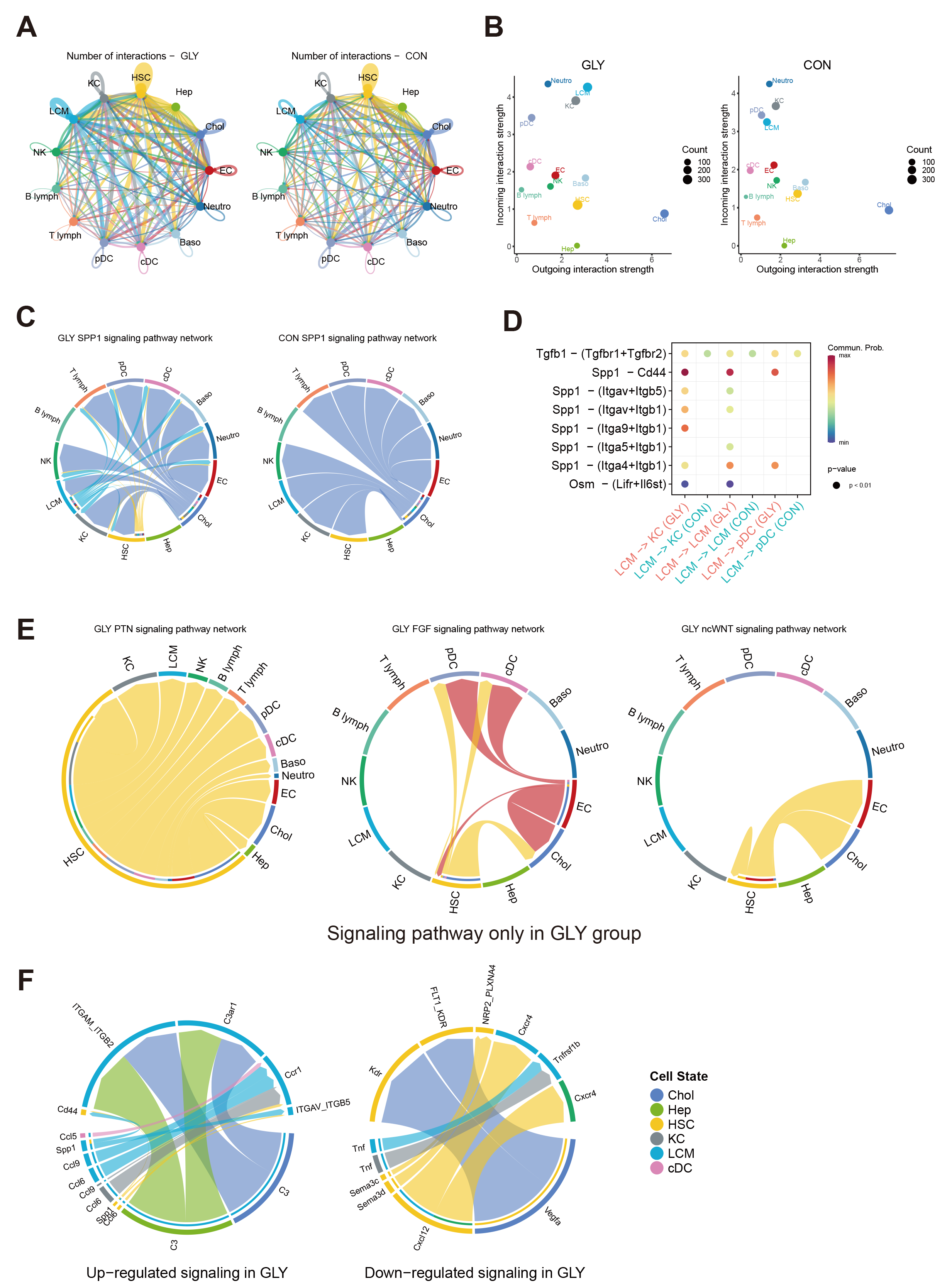


**Fig. S8
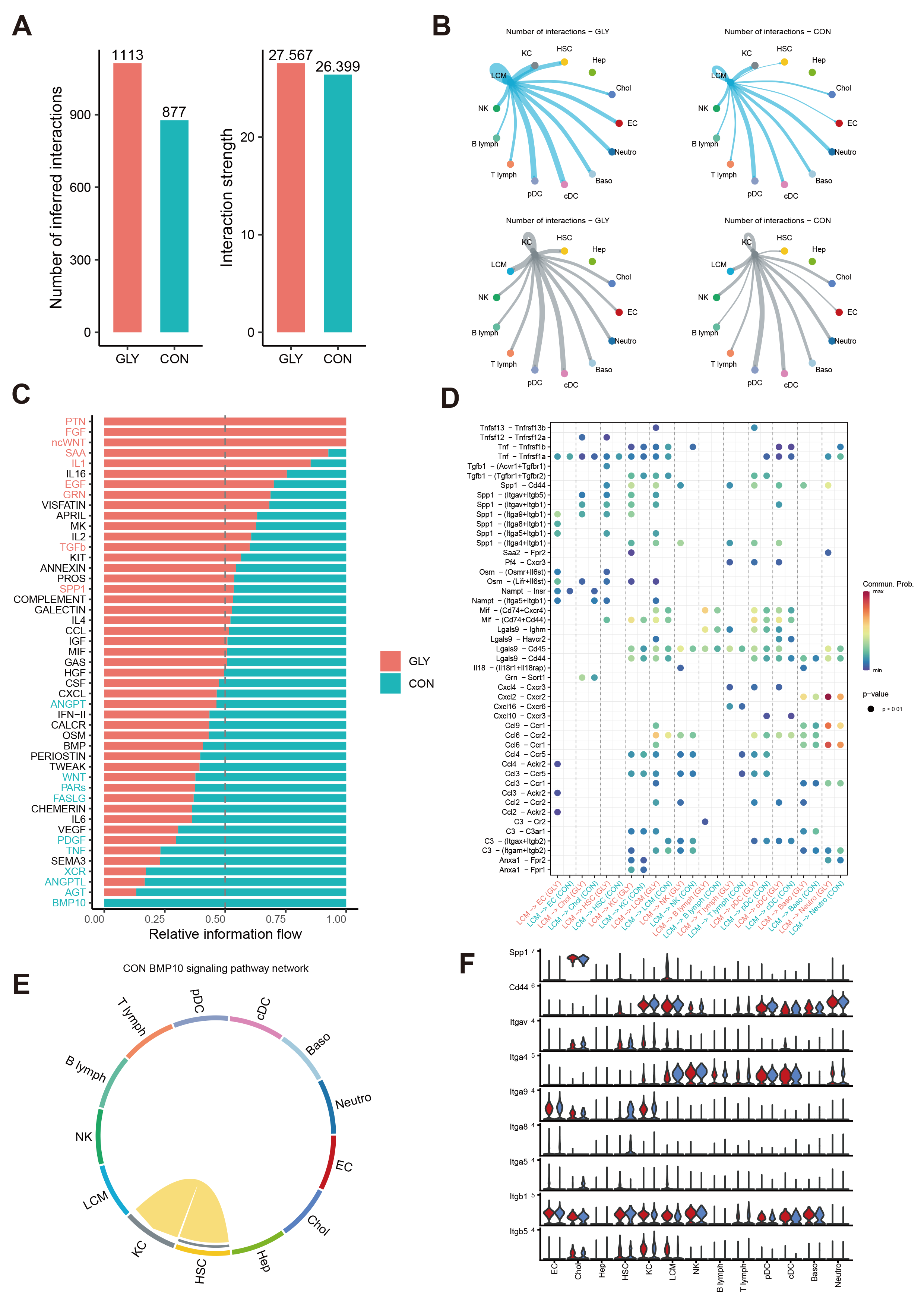
**

Supplement: Supplementary file 1 — Additional file 1: Fig. S1. Quality control for scRNA-seq. (A) Criteria for the degree of necrosis (left). Histopathological evaluation for all samples (right). (B) Consistency (nFeature_RNA, nCount_RNA, percent.mt) of cell capture and identification for each sample (left) and the two groups (right) after scRNA-seq data quality control. nFeature_RNA, nCount_RNA and percent.mt represent the number of genes, the number of transcripts and the percentage of mitochondrial genes of each cell, respectively. Fig. S2. Single-cell analysis of 13 major cell lineages. (A) UMAP showing all clusters from the control and GLY-treated groups. (B) Merged UMAP plot for all samples. (C) Heatmap of top 100 DEGs for major cell types. (D) Distribution comparison of the major cell clusters between the control and GLY-treated groups. (E) Histograms depicting the proportional changes of each cell type for individual samples (left) and the combined control and GLY-treated samples (right). Fig. S3. Single-cell analysis of HSCs. (A) UMAP showing all subclusters of HSCs from the control and GLY-treated groups. (B) Merged UMAP plot for all samples. (C) Proportional changes of each cell subtype for the two groups. (D) Pseudotime trajectory analysis implying the development of HSC subtypes. (E) Histogram displaying the proportional changes of the two groups under different states during the pseudotime analysis. (F) Violin plots showing the cell-cycle-related genes of each HSC subtypes. Fig. S4. Single-cell analysis of hepatocytes. (A) UMAP showing hepatocytes composed of three subclusters from the control and GLY-treated groups. (B) Merged UMAP plot for hepatocytes of all samples. (C) UMAP depicting the distribution of hepatocyte subclusters from the control and GLY-treated groups. (D) Histogram displaying the proportional changes of each subtype for every sample. (E) Boxplot of ROS score for the two groups. P value is from wilcox.test (unpaired and two-tailed); *P < 0.05. Fig. S5. Single-cell anal [file 11658_2023_426_MOESM1_ESM.docx]
